# Permeability Study of Polyphenols Derived from a Phenolic-Enriched *Hibiscus sabdariffa* Extract by UHPLC-ESI-UHR-Qq-TOF-MS

**DOI:** 10.3390/ijms160818396

**Published:** 2015-08-07

**Authors:** Isabel Borrás-Linares, María Herranz-López, Enrique Barrajón-Catalán, David Arráez-Román, Isabel González-Álvarez, Marival Bermejo, Alberto Fernández Gutiérrez, Vicente Micol, Antonio Segura-Carretero

**Affiliations:** 1 Department of Analytical Chemistry, Faculty of Sciences, University of Granada, Avda Fuentenueva s/n, 18071 Granada, Spain; E-Mails: iborras@ugr.es (I.B.-L.); albertof@ugr.es (A.F.G.); ansegura@ugr.es (A.S.-C.); 2 Research and Development Functional Food Centre, Health Science Technological Park, Avda Conocimiento s/n, 18016 Granada, Spain; 3 Instituto de Biología Molecular y Celular (IBMC), Miguel Hernández University, Elche, 03202 Alicante, Spain; E-Mails: mherranz@umh.es (M.H.-L.); e.barrajon@umh.es (E.B.-C.); vmicol@umh.es (V.M.); 4 Pharmacokinetics and Pharmaceutical Technology Area, Engineering Department, Universidad Miguel Hernández, San Juan de Alicante, 03550 Alicante, Spain; E-Mails: isabel.gonzalez@umh.es (I.G.-A.); mbermejo@umh.es (M.B.)

**Keywords:** *Hibiscus sabdariffa*, phenolic compounds, flavonoids, Caco-2 cells, UHPLC-ESI-UHR-Qq-TOF-MS

## Abstract

Previous findings on the capacity of *Hibiscus sabdariffa* (HS) polyphenols to ameliorate metabolic disturbances justify the necessity of studies oriented to find the potential metabolites responsible for such an effect. The present study examined the intestinal epithelial membrane permeability of polyphenols present in a phenolic-enriched *Hibiscus sabdariffa* extract (PEHS), free and encapsulated, using the Caco-2 cell line. Additionally, selected polyphenols (quercetin, quercetin-3-glucoside, quercetin-3-glucuronide, and *N*-feruloyltyramine) were also studied in the same absorption model. The powerful analytical platform used ultra-high-performance liquid chromatography coupled with ultra-high-resolution quadrupole time-of-flight mass spectrometry (UHPLC-ESI-UHR-Qq-TOF-MS), and enabled the characterization of seven new compounds in PEHS. In the permeation study, only a few compounds were able to cross the cell monolayer and the permeability was lower when the extract was in an encapsulated form. Pure compounds showed a moderate absorption in all cases. Nevertheless, these preliminary results may need further research to understand the complete absorption mechanism of Hibiscus polyphenols.

## 1. Introduction

*Hibiscus sabdariffa* L. (HS) (family Malvaceae), commonly named bissap, roselle, red sorrel, or karkade, is a tropical plant commonly used as a beverage in folk medicine for the treatment of diverse disorders such as constipation, heart ailments, high blood pressure, urinary tract infections, cancer, diabetes, or hepatic disorders [1,2,3,4]. Recent studies have focused on its antioxidant [5,6], antimicrobial [7,8], anti-inflammatory [9,10], anti-adipogenic [11], immunomodulatory [10], hepatoprotective [12,13], anticancer [14,15], cardioprotective [16,17,18], or diuretic activities [19]. Proteins, lipids, carbohydrates, minerals, and vitamins, as well as less polar compounds such as sterols, are present in its composition [20]. HS possesses organic and phenolic acids such as citric, hibiscus, or protocatechuic acids; flavonoids such as quercetin, luteolin, and their glycosides; and anthocyanins such as cyanidin-3-*O*-sambubioside, cyanidin-3-*O*-glucoside, or delphinidin-3-*O*-sambubioside, which are responsible for the bright red color of their calyces [11,21,22].

We have previously reported the capability of HS polyphenols to modulate triglyceride accumulation, oxidative stress, and inflammation in insulin-resistant adipocytes at relatively low doses, *i.e.*, 10–40 μg/mL (phenolic-enriched *Hibiscus sabdariffa* (PEHS) extract) and with large doses in human volunteers (10 g of an aqueous extract) [11,23], and to prevent fatty liver disease in a hyperlipidemic mouse model through miRNA-mediated glucose and lipid homeostasis regulation. We have accumulated enough evidence to propose that some flavonols may be the major contributors to the observed effects [17,24]. Nevertheless, a synergistic interaction among several components in the extract and a multi-targeted mechanism has been proposed [11,25]. Recent human studies in patients suffering metabolic syndrome support the potential therapeutic use of the extract derived from HS calyces against hypertension when administered at a dose of 125 mg/kg/day for four weeks [26]. In this scenario, the study of the absorption and bioavailability of the major compounds and the identification of the putative metabolites is needed to elucidate their molecular targets and for the complete understanding of their mechanism.

In the present study, the permeability of major polyphenols present in a PEHS, both free and encapsulated, has been studied for the first time using the Caco-2 human colon cell line as a model of human intestinal absorption. Additionally, the absorption of selected glycosylated and non-glycosylated polyphenols or metabolites related to the extract was also studied in the same model.

## 2. Results and Discussion

### 2.1. Characterization of the Phenolic-Enriched Hibiscus sabdariffa (PEHS) Extract

**Figure 1:**
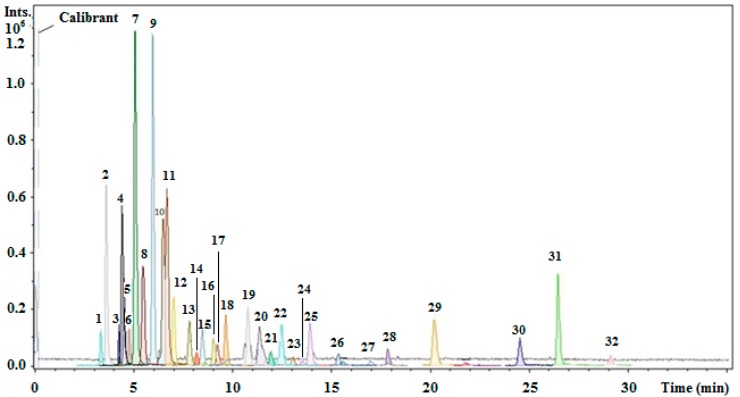
Base peak chromatogram of PEHS extract; peaks are identified with numbers according to the elution order.

The first step in the present study was to perform a complete characterization of the PEHS extract using an optimized analytical methodology. Thus, UHPLC-ESI-UHR-Qq-TOF-MS was used for the detection and characterization of phenolic and other polar compounds present in the extract. The base peak chromatogram obtained for this extract showed the presence of 32 compounds (Figure 1). These compounds were tentatively identified using the information provided by the UHR-Qq-TOF-MS, comparing their mass spectra and elution order with standards when available, and after a thorough survey of the literature. Data concerning the information of the detected compounds are summarized in Table 1, which lists the peak number, retention time, experimental *m*/*z*, calculated *m*/*z*, error (ppm) and sigma values, generated molecular formula, and proposed compound. Intraday and interday precisions were developed in order to assess the robustness of the method. A sample of the PEHS extract was injected three times in the same day (intraday precision, *n* = 3) and three times for three consecutive days (interday precision, *n* = 9). The intraday repeatability of peak area, expressed as the relative standard deviation (RSD) (%), was between the interval of 0.13%–2.87%, whereas the interday repeatability was between 0.37%–3.29%.

**Table 1:** List of the compounds detected in phenolic-enriched *Hibiscus sabdariffa* (PEHS) extract with the main details about mass-spectra analysis.

Peak Number	Retention Time (min)	*m*/*z* Experimental	*m*/*z* Calculated	Error (ppm)	Molecular Formula	Proposed Compound
1	3.4	207.0146	207.0146	−0.0	C_6_H_8_O_8_	Hydroxycitric acid
2	3.6	189.0041	189.0041	−0.1	C_6_H_6_O_7_	Hibiscus acid
3	4.3	235.0458	235.0459	0.4	C_8_H_12_O_8_	Hibiscus acid hydroxyethylesther
4	4.5	595.1303	595.1305	0.3	C_26_H_28_O_16_	Delphinidin-3-sambubioside
5	4.8	315.0720	315.0722	0.4	C_13_H_16_O_9_	Chlorogenic acid quinone
6	5.3	353.0874	353.0878	1.0	C_16_H1_8_O_9_	Neochlorogenic acid
7	5.5	579.1350	579.1355	0.9	C_26_H_28_O_15_	Cyanidin-3-sambubioside
8	6.0	217.0352	217.0354	1.0	C_8_H_10_O_7_	Hibiscus acid dimethylesther
9	6.3	353.0877	353.0878	0.3	C_16_H_18_O_9_	Chlorogenic acid
10	6.7	353.0877	353.0878	0.3	C_16_H_18_O_9_	Cryptochlorogenic acid
11	7.1	335.0408	335.0409	0.1	C_15_H_12_O_9_	Methyl digallate
12	7.9	369.0460	369.0463	0.8	C_15_H_14_O_11_	2-*O*-*trans*-caffeoyl-hydroxicitric acid
13	8.2	353.0874	353.0878	1.0	C_16_H_18_O_9_	1-*O*-caffeoylquinic acid
14	8.5	611.1246	611.1254	1.3	C_26_H_28_O_17_	Myricetin-3-arabinogalactoside
15	9.0	337.0925	337.0929	1.3	C_16_H_18_O_8_	Coumaroylquinic acid
16	9.2	236.0563	236.0564	0.6	C_11_H_11_NO_5_	UK
17	9.7	263.0772	263.0772	0.0	C_10_H_16_O_8_	Hibiscus acid hydroxyethyldimethylesther
18	10.7	595.1305	595.1305	−0.0	C_26_H_28_O_16_	Quercetin-3-sambubioside
19	11.3	335.0771	335.0772	0.4	C_16_H_16_O_8_	5-*O*-caffeoylshikimic acid
20	12.0	263.0773	263.0772	−0.3	C_10_H_16_O_8_	2-*O*-*trans*-feruloyl-hydroxicitric acid
21	12.3	609.1458	609.1461	0.5	C_27_H_30_O_16_	Quercetin-3-rutinoside
22	13.1	385.1139	385.1140	0.3	C_17_H_22_O_10_	UK
23	13.6	579.1356	579.1355	−0.2	C_26_H_28_O_15_	Kaempferol-3-*O*-sambubioside
24	14.0	463.0882	463.0882	0.0	C_21_H_20_O_12_	Quercetin-3-glucoside
25	15.3	593.1512	593.1512	−0.0	C_27_H_30_O_15_	Kaempferol-3-*O*-rutinoside
26	15.5	381.1190	381.1191	0.1	C_18_H_22_O_9_	Ethylchlorogenate
27	17.1	319.0826	319.0823	−0.8	C_16_H_16_O_7_	Methylepigallocatechin
28	17.7	457.1716	457.1715	−0.1	C_21_H_30_O_11_	UK
29	20.3	317.0309	317.0303	−1.8	C_15_H_10_O_8_	Myricetin
30	24.5	312.1245	312.1241	−1.2	C_18_H_19_NO_4_	*N*-feruloyltyramine
31	26.4	301.0358	301.0354	−1.4	C_15_H_10_O_7_	Quercetin
32	29.1	285.0415	285.0404	−3.6	C_15_H_10_O_6_	Kaempferol

Most of the identified compounds have been previously described in PEHS extract [11,22]. Nevertheless, seven new compounds were identified due to the use of UHR-Qq-TOF-MS, a detector with a high resolution and extreme sensitivity. Thus, peaks 3 and 26, proposed as hibiscus acid hydroxyethylesther and ethylchlorogenate, respectively, were detected in the previous analysis of the PEHS extract but it was impossible to confirm their identity with the available information. Furthermore, chlorogenic acid quinone, 1-*O*-caffeoylquinic acid, hibiscus acid hydroxyethyldimethylesther, 2-*O*-transferuloylhydroxicitric acid, and kaempferol, corresponding to peaks 5, 13, 17, 20 and 32, respectively, have been detected for the first time in the PEHS extract.

Moreover, other phenolic and polar compounds were identified in the extract, corresponding to different families. It should be pointed out that there was a presence of a wide variety of organic acids and derivatives, such as hydroxycitric and hibiscus acids, 2-*O*-*trans*-caffeoylhydroxycitric acid, hibiscus acid dimethylesther, hibiscus acid hydroxyethylesther, hibiscus acid hydroxyethyldimethylesther, 5-*O*-caffeoylshikimic acid, and coumaroylquinic acid. Several phenolic acids and derivatives were detected in the extract, such as chlorogenic acid, its isomers neo and cryptochlorogenic acids and derivatives such as chlorogenic acid quinone or ethylchlorogenate, as well as methyldigallate.

Furthermore, other flavonoids and their glycosidic derivatives were characterized in the extract. The presence of the following should be highlighted: quercetin and its glucoside, rutinoside, and sambubioside conjugates; kaempferol and its rutinoside and sambubioside forms; myricetin, myricetin-3-arabinogalactoside, and methylepigallocatechin. Additionally, anthocyanins responsible for the bright red color calyces of HS, specifically delphinidin-3-sambubioside and cyanidin-3-sambubioside, were also detected, as well as the compound *N*-feruloyltyramine.

Despite the efforts made in order to characterize the extract, Table 1 shows three compounds named as unknown (UK in Table 1) for which it was impossible to elucidate a structure due to lack of sufficient evidence. The table includes retention time, experimental *m*/*z*, molecular formulas, errors, and σ values.

The use of a powerful analytical technique such as UHPLC-ESI-UHR-Qq-TOF-MS has been a valuable tool, allowing the characterization of seven new compounds for the first time in this plant matrix. These new compounds were in their majority organic and phenolic acid derivatives, such as hibiscus acid hydroxyethylesther, ethyl chlorogenate, chlorogenic acid quinone, 1-*O*-caffeoylquinic acid, hibiscus acid hydroxyethyldimethylesther, and 2-*O*-*trans*-feruloyl-hydroxycitric acid. Furthermore, kaempferol was also detected in this extract for the first time. The compound ethyl chlorogenate has been found in various extracts of different plants with hepatoprotective and antiviral activities [27,28]. On the other hand, chlorogenic acid quinone is an oxidative product of chlorogenic acid by polyphenol oxidase, one of the major polyphenols present in plants [29,30]. The compound 1-*O*-caffeoylquinic acid has been found in different plants and foods, such as potato, tomato, or artichoke, among others [31,32,33]; also, the compound 2-*O*-*trans*-feruloyl-hydroxycitric acid is found in corn [34]. Moreover, kaempferol has been previously found in the petals of this plant matrix as well as in rat plasma after the oral administration of the PEHS extract [17,35].

### 2.2. Permeability of Free and Encapsulated PEHS Extracts

One of the aims of this study was to compare the intestinal absorption of HS compounds when the PEHS extract was in a free form or in an encapsulated formulation, using the Caco-2 model system. This model is a well-established *in vitro* model for the investigation of the intestinal permeability of different compounds or drugs [36,37]. Prior to the transport studies, the Caco-2 cell monolayer was established and validated. Monolayer integrity and reliability were ensured by measuring the transepithelial electrical resistance (TEER) values, which remained stable during the assay with the experimental conditions assayed. Afterwards, samples from apical and basolateral compartments obtained at different time points and cytoplasmic and membrane fractions of the cell monolayer collected at the end of the assay (see Section 3.3 and Section 3.4) were analyzed by UHPLC-ESI-UHR-Qq-TOF-MS in order to identify which compounds crossed the Caco-2 cell monolayer. The results are summarized in the Table 2.

**Table 2:** Compounds detected in the transport medium, cytoplasm, and membrane obtained in apical to basolateral (Ap-Bas) and basolateral to apical (Bas-Ap) transport directions in the Caco-2 assay with the free and encapsulated PEHS. The symbol + indicated that the compound was detected.

Extract	Free PEHS						Encapsulated PEHS					
Compound	Transport Medium		Cytoplasm		Membrane		Transport Medium		Cytoplasm		Membrane	
	Ap-Bas 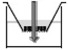	Bas-Ap 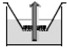	Ap-Bas 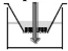	Bas-Ap 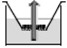	Ap-Bas 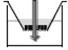	Bas-Ap 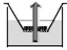	Ap-Bas 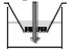	Bas-Ap 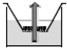	Ap-Bas 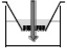	Bas-Ap 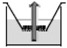	Ap-Bas 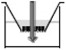	Bas-Ap 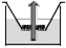
Hibiscus acid									+			
Neochlorogenic acid	+	+					+	+	+			
Hibiscus acid dimethylesther									+		+	+
Chlorogenic acid	+	+						+	+			
Cryptochlorogenic acid	+	+					+	+	+	+		
Methyldigallate		+						+	+			
Coumaroylquinic acid		+										+
5-*O*-caffeoylshikimic acid	+	+						+				
Methylepigallocatechin		+										
*N*-feruloyltyramine	+	+					+	+				
Quercetin	+	+	+		+				+			

Ap-Bas: Apical-Basolateral flux; Bas-Ap: Basolateral-Apical flux; +: Presence of the compound in the sample.

Thus, for the free PEHS extract, the analysis of the transport medium revealed the presence of chlorogenic acid, cryptochlorogenic acid, neochlorogenic acid, 5-*O*-caffeoylshikimic acid, *N*-feruloyltyramine, and quercetin in the receiver chamber in both flux directions. Additionally, the compounds methyldigallate, coumaroylquinic acid, and methylepigallocatechin were also detected only in the basolateral-apical flux.

Although the high polarity of the main compounds in PEHS suggested that encapsulation in liposomes would increase the permeability of these compounds, a lower permeability was observed when encapsulated PEHS was used. The number and amount of compounds transported across the membrane decreased compared to free PEHS extract. Thus, in the apical-basolateral flux study, the compounds detected in the transport medium of the receiver chamber were neochlorogenic and cryptochlorogenic acids and *N*-feruloyltyramine. On the contrary, more compounds were detected in the acceptor compartment for the basolateral-apical flow, such as chlorogenic acid and its isomers, methyldigallate, 5-*O*-caffeoylshikimic acid, and *N*-feruloyltyramine (Table 2).

These results showed the potential of the very sensible analytical instrumentation used, which allowed the detection of several compounds in the acceptor chambers in both flux directions and with the two PEHS assayed formulations.

The uptake of the compounds present in both formulations of PEHS by the Caco-2 cells was also monitored by analyzing the cytoplasmic content of the cell monolayer collected at the end of the assay. Quercetin was found in the cytoplasm of the Caco-2 cells when the permeation of the free extract was monitored in the apical-basolateral direction. On the other hand, in the case of the encapsulated form, cryptochlorogenic acid was the only compound detected in the cytoplasm of the cells in the basolateral-apical flux, while in the opposite direction, seven compounds were detected: hibiscus acid, hibiscus acid dimethylesther, chlorogenic acid and its isomers, methyldigallate, and quercetin.

Phenolic compounds have shown a significant capacity to bind phospholipid membranes [38,39,40] and this fact may account for most of their membrane-related effects [41]; therefore, the possible presence of the phenolic compounds from PEHS in the cell membrane fraction was also explored. Caco-2 cell membranes were collected at the end of the assay, treated as explained in the Section 3.3 and Section 3.4, and subsequently analyzed by UHPLC-ESI-UHR-Qq-TOF-MS. The results showed that only quercetin was detected to be associated with membranes in the apical-basolateral flow study for the free PEHS extract. On the other hand, for the encapsulated PEHS extract, hibiscus acid dimethylesther and coumaroylquinic acid were detected in the membrane fraction, although the last compound was only found in the basolateral-apical direction.

The fact that only a few compounds were able to pass across the gut barrier model in both permeation studies could be partly due to the complex composition of the extract. The presence of high concentrations of several polyphenols in the extract could lead to a saturation of the specific transport mechanisms of the phenolic compounds through the cell monolayer or to the loss of sink conditions. The results obtained here reveal valuable information in order to identify the putative metabolites that would reach the target tissues and would account for the effects of HS extract.

### 2.3. Permeation of Pure Compounds Related to PEHS Composition

Although the study of the absorption of a complex extract resembles the *in vivo* processes better, the interference among the different compounds and the saturation of membrane transporters makes it difficult to quantitate the absorption of the different compounds. To avoid this situation, we studied the absorption process of a selection of pure polyphenols related to PEHS, previously identified as metabolites in an animal model (quercetin, quercetin-3-glucoside, quercetin-3-glucuronide, and *N*-feruloyltyramine). Three different forms of the flavonol quercetin, *i.e.*, its aglycone, the glycosylated and the glucuronidated forms, were used in the absorption cell model, since the amount of quercetin derivatives found in PEHS was fairly abundant [11] and quercetin metabolites were detected in several mouse tissues [24] and plasma samples of rats [17] after the oral consumption of PEHS. *N*-feruloyltyramine was also studied since this compound was fairly abundant in PEHS extract and appeared as a metabolite in animal studies [17,24]. Neither phenolic acids nor anthocyanins were selected for absorption studies since phenolic acids have been thoroughly studied in the bibliography and anthocyanins are poorly absorbed [42,43,44,45,46].

Quercetin and *N*-feruloyltyramine showed significant absorption in the experiments performed with the whole extract, so their individual permeation study was of interest to understand their absorption in model conditions. There is a certain controversy in respect to the permeability of quercetin and its derivatives. Whereas the permeability of quercetin-glucoside conjugates was higher than that observed for the aglycone [47,48], the opposite conclusion was made in other assays [49]. Therefore, the glycosylated and aglycone forms were compared in the absorption cell model. On the other hand, quercetin-glucuronide conjugates have been postulated as major metabolites derived from the phase II metabolism of quercetin aglycone or different glycoside derivatives [50]. On some occasions, the metabolites generated inside the enterocytes could be transported again to the intestinal lumen by the ABC transporters.

Standard calibration curves of the compounds under study were prepared using apigenin at a concentration of 10 μg/mL as an internal standard. All calibration curves showed good linearity within different concentration ranges depending on the analytes studied. The limits of detection (LODs) and limits of quantification (LOQs) for individual compounds in standard solutions were also calculated as *S*/*N* = 3 and *S*/*N* = 10, respectively, where *S*/*N* is the signal to noise ratio (Table 3). The compound concentrations were subsequently determined using the corrected area of each individual compound (three replicates) and by interpolation in the corresponding calibration curve. Repeatability of the proposed method was measured as the relative standard deviation (RSD, %) in terms of concentration. A mixed solution containing the analytes was injected several times (*n* = 3) on the same day (intraday precision) and three times on three consecutive days (interday precision, *n* = 9). Intraday repeatability of the method developed for all the analytes was from 0.11% to 1.93%, whereas the interday repeatability ranged from 0.17% to 2.06%. The contents of the monitored compounds in the receiver chambers at different times are shown in Table 4.

In some cases, the analysis of the samples by UHPLC-ESI-UHR-Qq-TOF-MS collected at different times in the receptor compartment revealed that the compounds of interest were not detected. This happened in the samples collected after 30, 60 and 90 min for quercetin-3-glucuronide in the apical-basolateral flux. In other cases, the presence of these compounds was detected but could not be quantified since the contents were below the LOQs. Moreover, the presence of these compounds was not found in the analysis of the cytoplasm and membrane fractions collected at the end of the assay.

**Table 3:** Calibration data, where LOD is the limit of detection and LOQ is the limit of quantification.

Analyte	LOD (μg/mL)	LOQ (μg/mL)	Calibration Range (μg/mL)	Calibration Equations	*r*^2^
Quercetin	0.05	0.17	0.17–20	*y* = 13.372 *x* − 0.0038	0.998
Quercetin-3-glucoside	0.07	0.23	0.23–55	*y* = 2.5235 *x* + 0.0113	0.988
Quercetin-3-glucuronide	0.03	0.11	0.2–45	*y* = 11.545 *x* + 0.0087	0.992
*N*-feruloyltyramine	0.014	0.048	0.2–40	*y* = 14.995 *x* + 0.1088	0.990

**Table 4:** Concentration (μg/mL) of compounds from the receptor chamber in the individual permeability assays. Value = *X* ± SD. ND: non-detected, <LOQ: below the LOQ.

Apical-Basolateral Flux				
**Time (min)**	30	60	90	120
**Quercetin**	0.28 ± 0.02	0.49 ± 0.03	1.33 ± 0.02	1.32 ± 0.01
**Quercetin-3-glucoside**	<LOQ	<LOQ	<LOQ	0.47 ± 0.03
**Quercetin-3-glucuronide**	ND	ND	ND	<LOQ
***N*-feruloyltyramine**	0.69 ± 0.02	2.3 ± 0.2	4.2 ± 0.5	6.6 ± 0.8
**Basolateral-Apical Flux**				
**Time (min)**	30	30	30	30
**Quercetin**	1.1 ± 0.4	1.1 ± 0.4	1.1 ± 0.4	1.1 ± 0.4
**Quercetin-3-glucoside**	<LOQ	<LOQ	<LOQ	<LOQ
**Quercetin-3-glucuronide**	<LOQ	<LOQ	<LOQ	<LOQ
***N*-feruloyltyramine**	1.35 ± 0.04	1.35 ± 0.04	1.35 ± 0.04	1.35 ± 0.04

It is important to remark that the concentration found for all the compounds in the basolateral-apical direction was higher than that found in the flux of the other direction. Furthermore, the permeation of quercetin-3-glucoside and quercetin-3-glucuronide in the apical-basolateral direction occurred to such a lesser extent that these compounds could not be quantified for several time-points.

The maximum permeation of quercetin was reached after 90 min of the assay for both flux directions, but the final concentration in the basolateral-apical flux was around five-fold higher than the opposite flux (see Table 4). For the other assayed compounds, the maximum concentration was found in the samples collected at the end of the assay (120 min). These findings revealed a faster and more effective absorption of quercetin compared to their derivatives, which may justify the fact that the aglycone reaches its molecular targets in a more effective way [49].

In conclusion, our results suggest the existence of significant absorption across the intestinal barrier of some of HS polyphenols, most probably by passive diffusion, especially for quercetin and *N*-feruloyltyramine (compounds detected when the free and encapsulated PEHS extracts were assayed). With respect to *N*-feruloyltyramine, as far as we are concerned, this study represents the first report for the permeation study of this compound in the *in vitro* Caco-2 cell model. On the contrary, the results showed that the absorption of the glycosylated flavonols, *i.e.*, quercetin-3-glucoside and quercetin-3-glucuronide, occurred in to a lesser extent. Moreover, the higher permeability in the basolateral-apical direction found for all the assayed compounds suggests a secretion transport mechanism to the intestinal lumen, which produces a decrease in the net absorption of these compounds. Our results are also in agreement with those reporting a higher absorption of quercetin compared to its different glycosylated forms [49], and compared to those showing a higher permeability in the basolateral-apical direction [49,51].

## 3. Experimental Section

### 3.1. Chemicals and Reagents

All chemicals were of analytical HPLC grade and used as received. Ethanol, acetonitrile, soy phosphatidylcholine, formic acid, and trifluoroacetic acid were purchased from Sigma-Aldrich (St. Louis, MO, USA). The resin used for the preparative chromatography was AmberliteTM FPX66 (Rohm and Haas, Philadelphia, PA, USA). Standard compounds quercetin (purity ≥ 98%), quercetin-3-glucoside (purity ≥ 98%), and quercetin-3-glucuronide (purity ≥ 95%) were supplied by Sigma-Aldrich and *N*-feruloyltyramine (purity ≥ 95%) by Chem–Faces (Wuhan, China). Lastly, Hank’s Balance culture medium (HBSS) was obtained from Gibco (Life Technologies, Gaithersburg, MD, USA) and methanol and DMSO, used for the preparation of the stock solutions, were purchased from Fisher Scientific (Pittsburgh, PA, USA).

### 3.2. Preparation of PEHS

Primary aqueous extract was obtained from sun-dried calyces from plants harvested in Senegal, with an approximate plant-to-extract ratio of 5:1 as previously described [23]. The purified PEHS extract was kindly provided by Monteloeder, Inc. (Elche, Alicante, Spain) and was essentially prepared as previously described [11]. The retained phenolic fraction was finally eluted with 95% ethanol and 0.01% trifluoroacetic acid (TFA), rotatory evaporated and freeze-dried. The PEHS encapsulated formulation was performed using empty liposomes, whose final concentration was 2 mM, adding soy phosphatidylcholine to the blend. To permeabilize the liposome membrane ethanol 10% was used. The extract was dissolved in HBSS at the desired concentration (2500 μg/mL), stirred for 10 min, and finally filtered through 0.45 μm three times (PVD filter). 

### 3.3. Cell Culture and Permeability Studies

To assess the potential intestinal absorption of PEHS and pure compounds, experiments were conducted using Caco-2 cells derived from human colorectal adenocarcinoma cells (obtained from the American Type Culture Collection). Cells were cultured in Dulbecco’s Modified Eagle Medium (DMEM) containing 10% (*v*/*v*) fetal bovine serum (FBS) and incubated at 37 °C in a humidified 5% CO_2_ air atmosphere. Cell monolayers were prepared by seeding 5 × 10^5^ Caco-2cells/well on a six-well Transwell insert filter. Monolayers were used 19–22 days after seeding. The integrity of each cell monolayer was checked by measuring its transepithelial electrical resistance (TEER) with an epithelial voltohmmeter (EVOM, World Precision Instrument, Sarasota, FL, USA) before and after the experiments. The culture medium was replaced with fresh medium 24 h before the transport experiments. Compounds were added after washing the Caco-2 cell monolayer twice with pre-warmed HBSS medium (pH 7.4), in the apical or in the basolateral side, to evaluate the transport characteristics of the cells. Permeability studies were done by adding the PEHS solution at a concentration of 2500 μg/mL, quercetin at 18.1 μg/mL, quercetin-3-glucoside at 46.4 μg/mL, quercetin-3-glucuronide at 47.8 μg/mL, and *N*-feruloyltyramine at 31.3 μg/mL, while the receiving chamber contained the corresponding volume of transport medium. Samples were taken at different times (30, 60, 90, 120 min) and stored at −80 °C until their treatment.

After the permeation study, the Caco-2 cell monolayer was collected and the cells were lysed with three subsequent freezing-thawing cycles followed by bath sonication, of 10 min each step. Then, the samples were centrifuged during 15 min at 14,000 rpm and 4 °C, and the supernatants (cytoplasmic fraction) and the pellets (membranes) were stored at −80 °C.

### 3.4. Sample Treatments

For the UHPLC-ESI-UHR-Qq-TOF-MS analysis of the free PEHS, the extract was dissolved in ultrapure water up to a concentration of 2500 ppm, stirring in a vortex and filtered with single-use filter units of cellulose acetate (0.45 μm) prior to the injection into the HPLC system.

The samples collected in the receiving chamber during the permeability assays were centrifuged for 15 min at 12,000 rpm and 4 °C. The supernatant was spiked with apigenin stock solution, used as an internal standard at a concentration of 10 μg/mL, in order to avoid irreproducibility of results between analyses. Finally, the samples were stored at −80 °C until the analysis, avoiding possible degradations.

The cytoplasms and cell membranes collected at the end of the permeability assays were subjected to protein precipitation using methanol, vortex-mixed, kept at −20 °C for 2 h and centrifuged at 12,000 rpm for 15 min at 4 °C. Finally, the supernatants were evaporated in a vacuum concentrator, dissolved in 100 μL of methanol, and then spiked with 10 μg/mL of apigenin, used as an internal standard for their analysis.

### 3.5. Analytical Methodology

For the analysis of the samples, a Dionex Ultimate 3000 UHPLC (Thermo Scientific, Sunnyvale, CA, USA) was used coupled with an ultra-high resolution quadrupole time-of-flight mass spectrometer maXis (UHR-Qq-TOF-MS) from Bruker Daltonik (Bremen, Germany). The chromatographic separation was performed in a Zorbax Eclipse Plus C-18 column (4.6 × 150 mm, 1.8 μm) at a flow rate of 0.3 mL/min using an injection volume of 5 μL. The mobile phases consisted in water-acetonitrile (90:10, *v*/*v*) with 0.5% of formic acid as eluent A and acetonitrile as eluent B. The following multi-step linear gradient was applied: 0 min, 5% B; 10 min, 35% B; 32 min 75% B; 37 min, 5% B; finally, the initial conditions were held for three minutes to equilibrate the system before the subsequent injection.

The UHPLC system was directly coupled to a UHR-Qq-TOF mass spectrometer via an electrospray ionization interface (ESI) operating in negative ionization mode using a capillary voltage of +4.5 kV. The optimum parameters for the ionization source were: drying gas flow, 8 L/min; drying gas temperature, 200 °C; nebulizer gas pressure, 2 bar. The other optimum values for UHR-Qq-TOF-MS parameters were: funnel RF, 300.0 Vpp; collision RF, 400 Vpp; transfer time, 65 μs; pre-pulse storage, 6 μs; ion cooler, 40 Vpp.

For all the experiments the detection was carried out considering a mass range of 50–1100 *m*/*z*, and using nitrogen as nebulizing and drying gas. The instrument was calibrated externally with a 74900-00-05 Cole Palmer syringe pump (Vernon Hills, IL, USA) directly connected to the interface and containing a 10 mM sodium formate cluster solution. The calibration solution was prepared as follows: 10 μL of 1 M sodium hydroxide was mixed with 990 μL of 0.1% formic acid in water:isopropanol (1:1, *v*/*v*). The mixture was injected at the beginning of each run (Figure 1 calibration segment) and all the spectra were calibrated prior to compound characterization. Due to the compensation of temperature drifts in the instrument, this external calibration provided accurate mass values of better than 5 ppm. The accurate mass data of the molecular ions were processed using Data Analysis 4.0 software (Bruker Daltonik), which provides a list of possible elemental formulas via the Generate Molecular Formula Editor.

## 4. Conclusions

The use of a powerful analytical technique such as UHPLC-ESI-UHR-Qq-TOF-MS has been a valuable tool to carry out the absorption study of the phenolic compounds from a PEHS extract in an *in vitro* model for intestinal absorption. The proposed method allowed the characterization of seven new compounds for the first time in this plant matrix. These new compounds were in their majority organic and phenolic acid derivatives. In the permeability study of PEHS, only a few compounds were able to cross the cell monolayer in both flux directions. In addition, the results highlighted a lower permeation of the compounds from the encapsulated formulation of PEHS compared to the free formulation. Furthermore, four individual compounds related to PEHS extract were studied in the same absorption model (quercetin, quercetin-3-glucoside, quercetin-3-glucuronide, and *N*-feruloyltyramine), and the results indicated a significant absorption in the gut barrier model for all the compounds. On the other hand, all the assays showed a higher basolateral-apical permeability, suggesting the existence of a transport efflux mechanism of these compounds.
